# Amputation of an Extra-root with an Endodontic Lesion in an Invaginated Vital Maxillary Lateral Incisor: A Rare Case with Seven-year Follow-up

**DOI:** 10.7508/iej.2016.02.013

**Published:** 2016-03-20

**Authors:** Mehmet Kemal Çalışkan, Saeed Asgary, Uğur Tekin, Pelin Güneri

**Affiliations:** a * Department of Endodontics, Dental School, Ege University, İzmir, Turkey;*; b *Iranian Center for Endodontic Research (ICER), Research Institute of Dental Sciences, Dental School, Shahid Beheshti University of Medical Sciences, Tehran, Iran; *; c *Department of Oral and Maxillofacial Surgery, Dental School, Ege University, Izmir, Turkey; *; d *Department of Oral and Maxillofacial Radiology, Dental School, Ege University, Izmir, Turkey*

**Keywords:** Apical Periodontitis, Dens Invaginatus, Endodontic Therapy, Lateral Incisor, Periradicular Surgery

## Abstract

The developmental abnormality of tooth resulting from the infolding of enamel/dentin into the root is called dens invaginatus. Management of such cases is usually challenging due to the morphological complexity of root canal system. This report presents a rare treatment protocol of a clinical case of Oehler’s *type III* dens invaginatus combined with an endodontic lesion in a vital maxillary lateral incisor. Access to the endodontic lesion located between the central and lateral incisors was achieved by reflection of a full mucoperiosteal flap. Granulomatous tissue as well as aberrant root was removed and the surface of the root and adjacent coronal region were reshaped. Three years later, the patient was orthodontically treated. Seven years after completion of surgical/orthodontic management, the tooth remained asymptomatic and functional with normal periodontium/vital pulp. Radiographically, the healing of the lesion was observed. Actually, vitality of the invaginated tooth and communication between the invagination and the root canal were the most important factors in determining such minimally invasive treatment protocol. Depending on the anatomy of the root canal system, surgical amputation of an invaginated root can be performed to achieve a successful outcome in Oehler’s *type III* dens invaginatus cases, even though it is associated with apical periodontitis.

## Introduction

Malformations of teeth with variations involving either the crown or the root or both are described as dental hard tissue anomalies [1, 2]. Dens invaginatus is one of these anomalies, characterized with an infolding of enamel and dentin that may extend into the pulp cavity, into the root, and sometimes to the root apex [3]. It is a rather frequent anomaly with its frequency ranging from 0.04 to 10% [4]. 

Oehler classified dens invaginatus into three distinct types according to the depth of invagination and its communication with the periodontal ligament or periapical tissues; *Type I* is an enamel-lined minor invagination confined to the crown without extending beyond the cemento-enamel junction. *Type II* describes the extension of the invagination into the root, beyond the cemento-enamel junction ending in a blind sac. Some *type II* cases may communicate with the dental pulp. *Type III* includes penetration of the root by the invagination to form an additional apical or lateral foramen, usually without an immediate communication with the dental pulp [5, 6]. 

Based on the complex root canal anatomy and the shape of the pulp in the *type III* cases, treatment procedures are usually chosen from the following procedures: root canal treatment of the invagination only [7], endodontic therapy of main root canal and invaginated part [8], combined endodontic and surgical therapy [9], intentional replantation with retrograde surgery [10], and extraction [11].

The following unique case report describes a successful surgical aberrant root amputation of an invaginated extra-root with an endodontic lesion in a vital maxillary lateral incisor.

**Figure 1:**
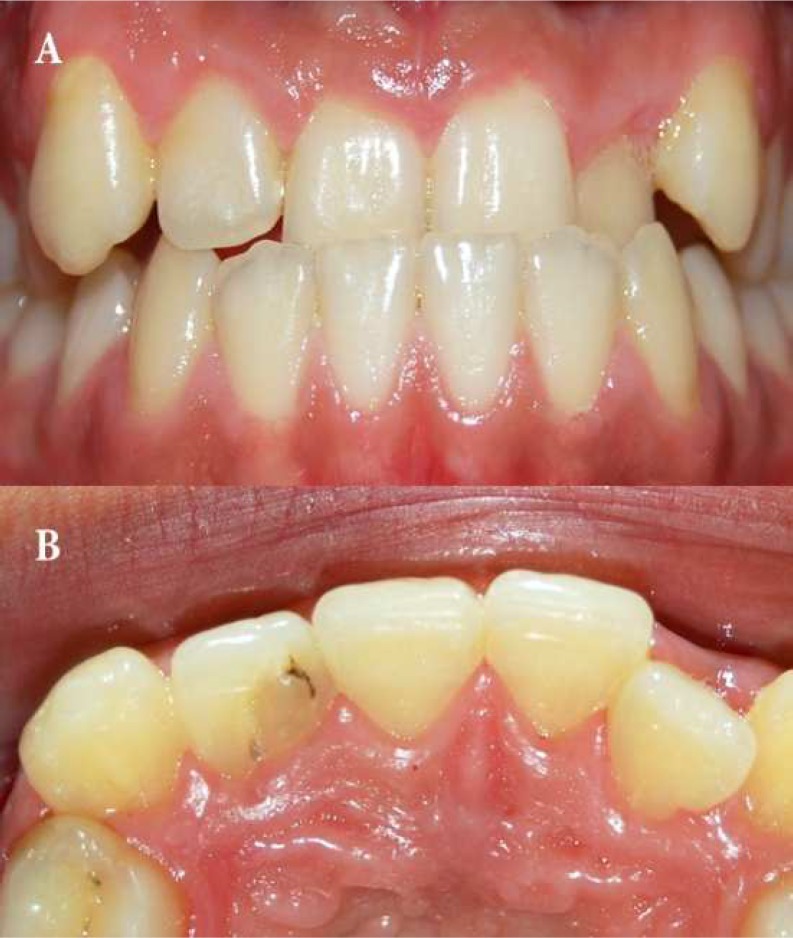
Clinical preoperative photographs of tooth #7; *A)* Labial view showing cross bite of maxillary central incisors and left lateral incisor and *B)* Palatal view of the same tooth

## Case Report

A 16-year-old male was referred to private clinic by a general dentist for evaluation and treatment of the maxillary right lateral incisor with enlarged clinical crown. The patient described moderate pain associated with the involved tooth. At the time of clinical evaluation, the patient’s symptoms had subsided. His medical history was not contributory and there was no history of trauma. The involved tooth was caries free and responsive to thermal and electrical pulp tests while the adjacent teeth were also responding. The clinical crown was larger than the contralateral tooth, especially the mesiodistal aspect. Crown appeared to be intact with a small palatal tuberculum without a pit or foramen coecum and revealed no discoloration, but mesial tilting of clinical crown with a slight depression on palatal surface near the mesial marginal ridge was evident (Figures 1A and B). No tenderness on percussion or palpation was noted, but the tooth had periodontal problem with gingival swelling combined with a pocket depth of 7 mm at the mesiopalatal surface communicating with the periradicular region. 

Radiographic examination revealed large periradicular radiolucency along the mesial surface adjacent to the mesial aspect of the tooth. There was a mesially directed radiopaque enamel-lined tract, which is related to the periapical radiolucency. Enamel lined tract was separated from the main root canal. The abnormal hard tissue structure of invaginated root was extending from the mesial aspect of the middle third of the main root canal to the cervical distal margin of the adjacent central incisor. The diagnosis was a vital tooth with *type III* dens invaginatus and associated chronic periradicular periodontitis. 

**Figure 2:**
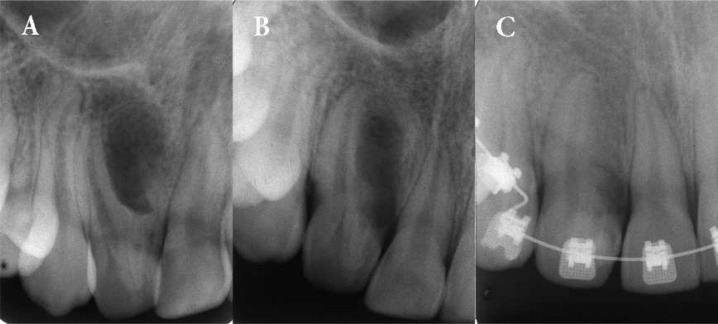
*A)* Preoperative radiograph showing the tooth #7 with severe *type III* dens invaginatus with a large periradicular lesion; *B)* Ten days after surgical amputation of invaginated aberrant root; *C)* Four-year radiographic recall of involved tooth during an orthodontic treatment. Periapical radiograph showing complete healing of the periradicular lesion and the asymptomatic tooth responded a positive response to cold test

The treatment plan included the surgical amputation of bucally-located anomalous root-like structure of dens invaginatus. Under local anesthesia, a full mucoperiosteal flap was elevated; access to the pathological area was obtained between left central and lateral incisors. The lesion was curetted and the specimen was sent for histopathological examination. The abnormal hard tissue structure of invaginated root with groove was removed using a tapered diamond fissure and finishing burs under continuous irrigation with saline solution. The surface of the main root adjacent to the cervical region was reshaped and smoothed while exposure of the dentinal tubules was minimized. The flap was replaced and sutured (Figures 2A and B). The palatal coronal surface near the mesial marginal ridge was restored with composite resin (Filtek Z250, 3M ESPE, St. Paul, MN, USA). 

**Figure 3.**
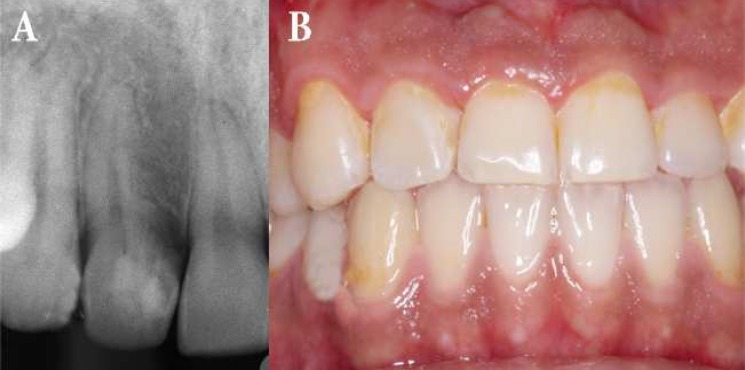
Seven-years after completion of surgical/orthodontic treatment. *A)* Periapical radiography showing complete bone healing. The pulp of the asymptomatic tooth continued to provide positive responses to pulp-sensibility testing and periodontal probing depths were within normal limits, *B)* Facial clinical appearance of the case

**Figure 4:**
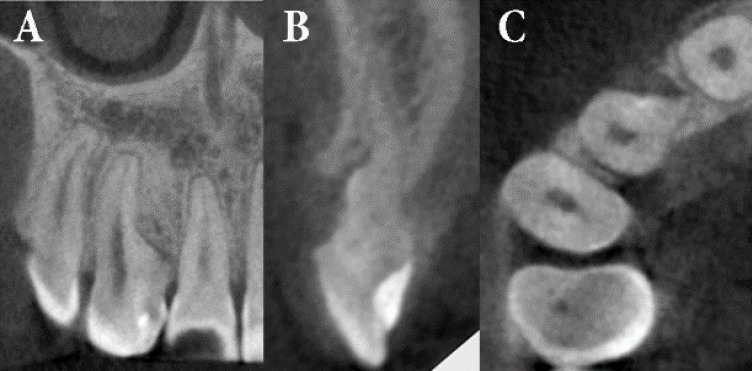
*A)* No bone defect within the operation area in coronal CBCT; *B)* On the sagittal image, the trabecular bone over the root surface is apparent, the operation area seems to be filled with gingiva since removed root piece was located at the buccal area. Clinical examination of the related area revealed no pathological periodontal pocket; *C)* The overhanging tooth structure was elongated mesially towards the distal in the axial view

The histologic diagnosis of the biopsy material was granulation tissue with severe chronic inflammation. A postoperative four-year periapical radiography taken during orthodontic treatment showed resolution of the periradicular radiolucency while the tooth remained asymptomatic and continued to give a positive response to cold test. Periodontal probing depths were found to be within normal limits (Figure 2C). Seven-year follow-up radiographies (Figures 3 A and B) and CBCT images (Figures 4A to C) of the involved tooth revealed the absence of periradicular lesion and the patient remained asymptomatic.

## Discussion

Treatment of dens invaginatus is undoubtedly an endodontic challenge, especially because of the complicated morphology of tooth and the complexity of associated root canal system. It is therefore important to identify the morphologic character of the canal before treating a tooth with dens invaginatus associated with pulp pathosis [4].

In cases of Oehlers’ *type III* dens invaginatus, the pulp is confined within the wall of the invagination process, there is usually no communication between the root canal and the invagination itself. Although the invagination may seem to be completely lined by enamel, the apical portion of the invagination is more often lined only by cementum [5]. Bacterial invasion proceeds along the invagination tract to the deep surrounding tissues exiting by an apical or lateral opening, leading to the development of periradicular pathosis. In such teeth, the pulp may be bypassed and remain vital, unless apical vessels become secondarily involved [6]. 

Following eruption, teeth with dens invaginatus, lose the main blood supply of the connective tissue in the invaginated space which subsequently results in necrosis of the tissue within the invaginated tract and this in turn, leads to the development of periradicular inflammation [12] and the presence of infection in the invagination could delay root development [13].

The presence of communication between the invagination and the pulp may be an important prognostic value, but in the present case, it was presumed that such communication does not exist. On the other hand, communication between the invagination and the oral environment was not apparent clinically, but the abnormal shape of an invaginated root caused a plaque-retentive area prompting the development of a localized advanced periodontitis that may lead to occurrence of periradicular lesion.

Researchers suggested that pulp necrosis and periradicular pathosis may occur in permanent incisors with abnormal crown or incisors with root groove defect [14, 15]. Furthermore, the palatogingival groove is characterized by recurrent periodontal or acute alveolar abscess, dental mobility and gingival recession resulting from an infrabony periodontal defect at the site of the groove. Most moderate and complex palatogingival grooves manifest themselves as a long-standing pulpo-periodontal disease [16]. Surgical treatment should be considered in *type III* dens invaginatus with endodontic failure or in teeth, which cannot be treated non-surgically because of anatomical problems or failure to gain access to entire root canal system [17]. 

Despite the complex anatomy of the *type III* dens invaginatus besides the diagnosis of periradicular lesion, pulp health was retained after endodontic treatment [18] or combined endodontic and surgical therapy of an invaginated root canal alone [9]. To the best of our knowledge, this is the unique first report demonstrated an invaginated root amputation to maintain the pulp vitality of the main root. 

## Conclusion

The present case report describes the successful surgical treatment of an extra-root of a *type III* dens invaginatus associated with an apical lesion without interfering with the vitality of the main root.
